# Reversal of Endometriosis-Induced Unilateral Ureteral Obstruction and Hydronephrosis With Medical Therapy

**DOI:** 10.7759/cureus.73983

**Published:** 2024-11-19

**Authors:** Tai Nguyen, Martin Nguyen, Thao Hoang

**Affiliations:** 1 Obstetrics and Gynecology, Hung Vuong Hospital, Ho Chi Minh, VNM; 2 Urology, West Virginia School of Osteopathic Medicine, Lewisburg, USA

**Keywords:** deep infiltrating endometriosis, endometriosis, hydronephrosis, ureteral endometriosis, ureteral obstruction

## Abstract

Endometriosis is a common condition among women of reproductive age worldwide, with the urinary tract being the second most frequently affected extragenital organ system, particularly the bladder and ureters. Ureteral endometriosis (UE) is relatively rare, often asymptomatic, and can lead to progressive renal function loss if not addressed promptly. Early diagnosis and intervention are essential, requiring a high index of suspicion. We present a case of UE in a patient who experienced moderate dysmenorrhea and back pain. Imaging revealed left-sided moderate hydronephrosis and ureteral dilation, suggestive of endometriosis. After a comprehensive consultation, the patient was treated with goserelin. Following three months of regular monitoring, the hydronephrosis and ureteral dilation resolved. She then transitioned to dienogest for six months, followed by the insertion of a levonorgestrel-releasing intrauterine system. This case highlights the effectiveness of hormonal treatments in managing UE, particularly in patients with mild to moderate symptoms who aim to preserve fertility.

## Introduction

Endometriosis is a chronic condition characterized by the ectopic growth of endometrial-like tissue outside the uterine cavity, affecting 5-10% of women of reproductive age [1]. It is a debilitating disease, commonly presenting with symptoms such as chronic pelvic pain and infertility [2]. The most common types of endometriosis include superficial peritoneal lesions, deep infiltrating endometriosis (DIE), and endometriomas [3,4]. DIE is defined as the infiltration of endometriotic lesions into the peritoneum deeper than 5 mm [5]. The prevalence of DIE is estimated at 1-2%, and 95% of endometriosis cases are associated with severe pain [6].

Endometriosis most often affects the ovaries and pelvic peritoneum. Genitourinary involvement occurs in 1-2% of cases, with the bladder and ureters being the most commonly affected sites [7,8]. Among urinary tract involvement, the bladder is most frequently affected (85%), followed by the ureters (10%), kidneys (4%), and urethra (2%) [8]. Ureteral endometriosis (UE), a rare condition with a prevalence of 0.1%, is associated with a significant risk of renal function loss, with 25-50% of kidney function typically compromised by the time of diagnosis [7]. UE can be classified into intrinsic and extrinsic types, depending on whether the ectopic endometrial tissue invades the ureters directly or compresses them externally [7]. Due to diagnostic challenges and the often silent progression of renal impairment, UE can result in significant morbidity [8].

## Case presentation

Our patient was a 38-year-old female (Gravida 2, Para 2) in her late 30s who presented to our hospital with a history of moderate dysmenorrhea and moderate left back pain. She had attempted over-the-counter ibuprofen, but the pain was unresponsive. Her BMI was 17.0 kg/m², and her blood pressure was 125/90 mmHg; other vital signs were normal. Clinical examination revealed normal bowel sounds and no abdominal tenderness on palpation. A bimanual examination revealed an adnexal mass measuring approximately 8 × 6 × 7 cm, which was firm, immobile, and located in the cul-de-sac with multiple nodules.

**Figure 1:**
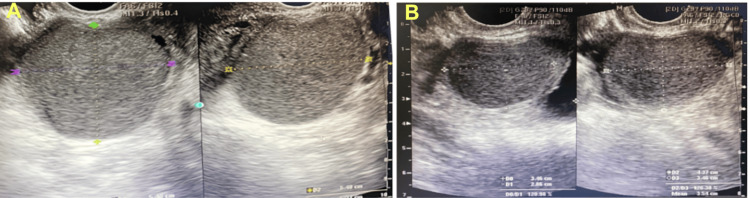
Transvaginal ultrasound findings: (A) A hypoechoic mass (54 × 53 × 54 mm) in the left ovary prior to medical therapy; (B) A hypoechoic mass (45 × 35 × 29 mm) in the right ovary prior to medical therapy

**Figure 2:**
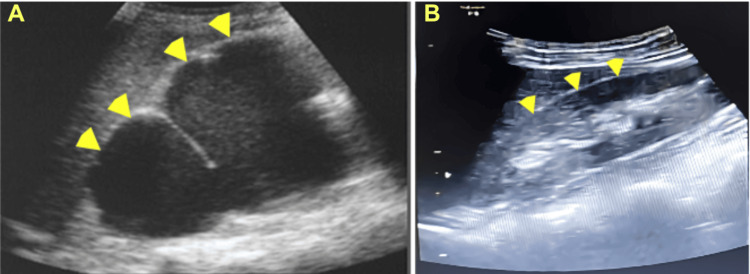
Transabdominal ultrasound findings: (A) Hydronephrosis of the left kidney upon presentation (yellow arrowheads); (B) Resolution of left hydronephrosis after three months of goserelin therapy (yellow arrowheads)

She had been transferred from her local hospital with a diagnosis of adenomyosis, uterine leiomyoma, and dysmenorrhea. A hysterectomy had been suggested, but she strongly desired to preserve her uterus for future pregnancies and sought a second opinion. Her complete blood count and basic metabolic panel were normal, but her CA-125 level was elevated at 144 IU/mL. Her estimated GFR (eGFR) was 61.8 mL/min, prompting an abdominal ultrasound to investigate both the urinary tract and ovaries. Transabdominal and transvaginal ultrasound findings demonstrated endometriosis involving both ovaries and the pouch of Douglas, as well as adenomyosis in the posterior uterine wall. Left and right endometriomas were noted (54 × 53 × 54 mm and 45 × 35 × 29 mm, respectively) (Figure 1). Additionally, moderate to severe left hydronephrosis and left ureteral dilation (0.8 cm) were reported (Figure 2), with the obstruction located at the distal third of the left ureter. Subsequently, CT confirmed the ureteral obstruction due to DIE involving the lower part of the left ureter. Ureteroscopy revealed an external mass compressing the left side of the bladder, causing a dislocation of the left ureteral orifice. The guidewire could not pass beyond 6 cm due to the external obstruction, while the right ureter appeared normal.

**Figure 3:**
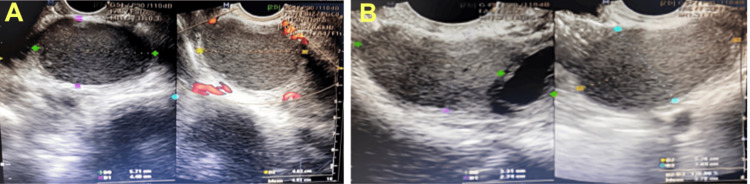
Transvaginal ultrasound findings: (A) A hypoechoic mass (44 × 57 × 46 mm) in the left ovary after three months of goserelin therapy; (B) A hypoechoic mass (45 × 33 × 27 mm) in the right ovary after three months of goserelin therapy

Following clinical consultation with the urology and nephrology departments, a treatment plan was initiated. Goserelin was prescribed at a dose of 3.6 mg subcutaneously (SC) every four weeks for a total of three doses. Afterward, the left and right endometriomas measured 44 × 57 × 46 mm and 45 × 33 × 27 mm, respectively (Figure 3). Hydronephrosis resolved on transabdominal ultrasound (Figure 2). Dienogest (DNG), an oral progestin, was started at a dose of 2 mg/day for six months. In the first two weeks, the patient experienced mild abnormal uterine bleeding, which decreased thereafter. Eventually, the patient was switched to a levonorgestrel-releasing intrauterine system (LNG-IUS) due to the cost of long-term DNG. She tolerated the treatment well and did not report any side effects related to hypoestrogenic states, including vasomotor symptoms, vaginal atrophy, dyspareunia, or mood instability. No remarkable findings were detected on ultrasound during follow-up.

## Discussion

The incidence of endometriosis with urinary tract involvement is highly variable, largely due to the limited current literature on this topic [8]. These cases account for approximately 0.01-2% of all endometriosis cases, with an average age of 30-35 years [8]. In a case series of 1054 patients, Vercellini et al. [9] reported six cases of UE, all involving the left ureter. In their literature review, the same group found that 64% of UE cases affected the left side, a significant difference compared to the right. They suggested that the asymmetry of both ureters and the differences between the left and right hemipelvis may promote the adhesion and growth of endometrial tissue on the left pelvic wall [9]. The most common presentation of UE is unilateral obstruction of the distal third of the ureter. Associated pathologies include endometriomas (52-68%), uterosacral ligament involvement (50%), and bowel involvement (39%) [8,10]. Clinical presentations can vary, with patients potentially presenting with flank pain, hematuria, unexplained hypertension, dysmenorrhea, dyspareunia, and menorrhagia [11,12]. UE should be suspected if a pelvic mass is palpated during physical examination in a relevant clinical context [11]. However, up to 50% of cases may be asymptomatic, leading to delayed diagnosis and silent loss of kidney function [10,12]. Other, less common presentations of UE may include bilateral obstruction or unilateral ureteral obstruction with hypertension [13-15]. There are two major types of UE: extrinsic (80%) and intrinsic (20%). Extrinsic UE occurs when endometrial tissue invades the submucosa and adventitia of the ureter, while intrinsic UE affects the uroepithelial and submucosal layers [16].

Undetected UE may increase the risk of recurrent UTIs, pyelonephritis, and irreversible renal failure [11]. Therefore, prompt diagnosis and effective treatment are crucial. Currently, there are no established guidelines or consensus on the optimal diagnostic approach for UE. However, physical signs such as uterosacral ligament nodularity or involvement of the pouch of Douglas may suggest DIE and its potential impact on the urinary tract [11].

Diagnostic tests for UE may include ultrasound, laparoscopy, CT, MRI, excretory urography, and ureteroscopy with endoluminal ultrasound [12]. Ultrasound and MRI are particularly useful for assessing the extent of the disease and for follow-up purposes [11]. Ultrasound is the first-line modality for evaluating patients with urinary symptoms [17]. Routine use of ultrasound to rule out ureteral obstruction is recommended, as many UE cases are incidentally diagnosed [12]. Additionally, periodic ultrasounds every six months can help detect early obstructions and guide prompt treatment if needed. In a meta-analysis of 11 studies on the accuracy of transvaginal ultrasound, Guerriero et al. [18] reported that ultrasound had a sensitivity of 62% (95% CI, 40-80%) and a specificity of 100% (95% CI, 97-100%) for detecting bladder endometriosis in patients with suspected DIE. Similarly, Pateman et al. [19] conducted a study of 848 women with chronic pelvic pain, following them for over 14 months. They found a sensitivity of 92% (95% CI, 63.9-99.8%) and a specificity of 100% (95% CI, 97.6-100%) when using ultrasound to detect UE. These findings emphasize the critical role of ultrasound in detecting urinary tract involvement in suspected endometriosis cases [17,19]. It is also recommended that routine ultrasound be performed in both known and suspected endometriosis cases to investigate the entire urinary tract and facilitate the early detection of asymptomatic hydronephrosis caused by ureteral obstruction [19]. Timely intervention can help prevent the silent loss of kidney function.

CT is useful in assessing extrinsic UE, particularly regarding the extent of disease and the severity of obstruction [20]. However, CT is not recommended for evaluating exclusively intrinsic UE [12] and is not helpful in mapping urinary tract endometriosis. Nonetheless, CT is often one of the first imaging modalities used in patients with non-specific abdominopelvic complaints [17]. If a soft tissue mass is identified on the ureters, further investigation with ultrasound or MRI is recommended to explore potential endometriosis involving the urinary tract [17].

MRI offers better sensitivity and specificity for extrinsic UE compared to intrinsic UE, with the ability to detect extrinsic lesions in over 80% of cases [21]. Laparoscopy is another reasonable diagnostic option for extrinsic UE, allowing direct visualization of endometrial lesions around the ureters, supported by multiple studies [22,23]. MRI and laparoscopy each have distinct advantages and limitations. MRI can complement laparoscopy by providing a more comprehensive view of the entire ureteral course in the pelvis, especially in severe cases with significant adhesions that may limit the laparoscopic exploration. The choice of modality depends on the specific information needed for diagnosis and intervention.

Our patient presented with moderate dysmenorrhea, and an initial decrease in eGFR (61.8 mL/min) suggested a urinary tract issue. Subsequently, an ultrasound revealed left hydronephrosis and left ureteral dilation. Given the presence of DIE, bilateral ovarian endometriomas, and these renal findings, UE was highly suspected. CT imaging further confirmed left hydronephrosis and left ureteral dilation.

The treatment goals for UE include relieving obstruction to preserve renal function and providing symptomatic relief. Management strategies vary based on the extent of the disease and the severity of renal impairment, requiring a tailored approach for each patient due to the diverse presentations of UE. Treatment options may involve hormonal therapy, surgical intervention, or a combination of both [12,24,25]. The primary aim of medical therapy is pain relief, as pharmacologic treatments are not cytoreductive; endometriotic lesions may recur and regrow after treatment cessation [25]. While surgery is often considered the standard approach for UE, medical therapy is sometimes used as adjunctive treatment to prepare patients for surgery [6,8,24]. In cases managed with hormonal therapy, regular follow-up is essential [8,12]. Hormonal treatments work by inhibiting ovulation, reducing menstruation, and optimizing the hormonal environment to suppress the growth of ectopic endometriotic tissue [25].

In our case, the patient had several reasons to consider a trial course of medical therapy. First, she strongly desired to preserve her uterus for future pregnancies. Second, she preferred to avoid surgical interventions whenever possible. Third, she denied symptoms of oliguria or dysuria, and her vital signs were normal. After thorough consultation with urologists and ICU physicians, the team decided to initiate medical therapy with goserelin and DNG, with close follow-up (one to two times per month) to regularly assess renal function. Goserelin was administered at a dose of 3.6 mg SC every four weeks for a total of three doses. At the same time, DNG was prescribed at 2 mg per day orally. Gonadotropin-releasing hormone (GnRH) agonists have been proven to be effective in managing pelvic pain associated with endometriosis [26]. Although these treatments can cause hypoestrogenic side effects such as vasomotor symptoms, vaginal atrophy, and mood instability, we considered the benefits to outweigh the risks in this case.

After three months, there was a significant improvement in the patient’s eGFR (from 61.8 mL/min to 90.1 mL/min), serum creatinine (from 1.3 mg/dL to 0.8 mg/dL), and CA-125 (from 144 IU/mL to 67 IU/mL). Follow-up ultrasound showed marked improvement in the left hydronephrosis, with no further evidence of hydronephrosis or ureteral dilation. During subsequent check-ups, she reported no symptoms of hot flashes, dyspareunia, or mood instability. These results align with findings from various case reports involving the use of progestin or GnRH agonists for managing UE [24,27].

In a case series by Rivlin et al., three patients with UE were treated with leuprolide acetate, a GnRH agonist, with favorable results [24]. Two of the patients had unilateral UE, and one had bilateral UE. Leuprolide successfully relieved ureteral obstruction in two out of three cases (66.7%), although it was ineffective in one case of intrinsic unilateral UE. The treatment period ranged from six to nine months. Similarly, Gantt et al. [27] reported successful reversal of ureteral obstruction due to pelvic endometriosis with progestin therapy.

For our patient, DNG was continued for a total of six months. Given the high cost of DNG, the patient was advised to switch to an LNG-IUS. She is currently being followed up every six months, and she expressed a desire for another pregnancy next year. As a result, the LNG-IUS is planned to be removed soon. Consultant urologists have decided not to pursue further interventions at this time.

Isolated cases of successful treatment of UE with danazol, a synthetic derivative of 17-ethynyl testosterone, have been reported [28,29]. However, there are also instances where treatment with both danazol and aromatase inhibitors has failed [30,31]. In cases where dense fibrotic tissues have formed, response to hypoestrogenic drugs tends to be poor, making hormonal therapy unlikely to provide effective relief of ureteral obstruction in advanced cases of UE [8,29]. Notably, even in cases that respond to medical therapy, it is expected that ureteral obstruction may recur once treatment is discontinued [8].

Surgical intervention is indicated in advanced cases of UE with extensive inflammation and fibrosis, as scar tissue is not responsive to medical therapy. Surgery is often required in the most severe cases of UE and can be performed via traditional laparotomy or minimally invasive procedures. The typical surgical management of UE includes a hysterectomy with bilateral salpingo-oophorectomy, with or without nephrectomy. One of the main objectives of surgery is to remove the primary source of estrogen production, thereby preventing the further growth of ectopic endometrial tissue. If renal function can be preserved, ureterolysis is recommended for extrinsic UE. For intrinsic UE, resection of the affected segment followed by ureteroureterostomy is necessary [12]. Postoperative follow-up is essential to monitor for potential ureteral stricture or deterioration of renal function. Some authors also suggest a combined approach in appropriate cases, starting with a trial of medical therapy before proceeding to surgery [12,24].

## Conclusions

UE is a rare condition with diverse clinical presentations, and up to half of the cases may be asymptomatic, leading to a gradual, unnoticed loss of kidney function. Therefore, clinicians must maintain a high level of suspicion, particularly in patients with features suggestive of DIE. Some studies recommend a routine ultrasound to assess the entire urinary tract in both suspected and confirmed cases of endometriosis, given the high incidence of asymptomatic cases. We presented a case of UE where the ureteral obstruction was successfully reversed after three months of treatment with goserelin, DNG, and LNG-IUS. While surgical intervention is required for severe cases of UE, medical therapy may be an appropriate option for well-selected patients, provided there is close monitoring of renal function and ureteral obstruction. Imaging modalities such as ultrasound and MRI are valuable for mapping urinary tract endometriosis. A multidisciplinary approach involving specialties such as radiology, gynecology, and urology is critical to optimize the management of these patients.
